# Serum-Induced Keratinization Processes in an Immortalized Human Meibomian Gland Epithelial Cell Line

**DOI:** 10.1371/journal.pone.0128096

**Published:** 2015-06-04

**Authors:** Ulrike Hampel, Antje Schröder, Todd Mitchell, Simon Brown, Peta Snikeris, Fabian Garreis, Carolina Kunnen, Mark Willcox, Friedrich Paulsen

**Affiliations:** 1 Department of Anatomy II, Friedrich-Alexander-University Erlangen Nürnberg, Erlangen, Germany; 2 Illawara Health and Medical Research Institute and School of Medicine, University of Wollongong, Wollongong, New South Wales, Australia; 3 School of Optometry and Vision Science, University of New South Wales, Sydney, New South Wales, Australia; Inserm U995-Université de Lille, FRANCE

## Abstract

**Purpose:**

The aim of this study was to evaluate a human meibomian gland epithelial cell line (HMGEC) as a model for meibomian gland (patho)physiology *in vitro*.

**Methods:**

HMGEC were cultured in the absence or presence of serum. Sudan III lipid staining, ultrastructural analysis and lipidomic analyses were performed. Impedance sensing, desmoplakin 1/2 mRNA and cytokeratin (CK) 1, 5, 6, 14 levels were evaluated. Serum containing medium supplemented with higher serum, glucose, an omega-3 lipid cocktail, eicosapentaenoic acid or sebomed medium were investigated for lipid accumulation and ultrastructural morphology.

**Results:**

Lipid droplet accumulation in HMGEC was induced by serum containing media after 1 day, but decreased over time. Cultivation in serum induced desmosome and cytokeratin filament formation. Desmoplakin 1/2 gene levels were significantly upregulated after 1d of serum treatment. Furthermore, the normalized impedance increased significantly. Lipidome analysis revealed high levels of phospholipids (over 50%), but very low levels of wax ester and cholesteryl esters (under 1%). Stimulation with eicosapentaenoic acid increased lipid accumulation after one day.

**Conclusion:**

Serum treatment of HMGEC caused lipid droplet formation to some extent but also induced keratinization. The cells did not produce typical meibum lipids under these growth conditions. HMGEC are well suited to study (hyper)keratinization processes of meibomian gland epithelial cells *in vitro*.

## Introduction

Meibomian gland dysfunction came into focus because most patients suffering from dry eye disease have abnormal meibomian gland function [1]. The meibomian gland is a holocrine sebaceous gland that produces meibum, an oily secretion that forms the outer layer of the tear film. The meibomian gland consists of basal proliferating cells that show no lipid accumulation whereas during ascent through different stages of maturation cells accumulate lipids progressively until the cell ruptures. After disintegration of mature meibocytes, the released cytoplasm, cell membranes and accumulated lipids form the meibum. The recent development of a meibomian gland epithelial cell line (HMGEC) enables the physiology and pathophysiology of meibocytes to be studied *in vitro* [2]. However, the method for handling and cultivation of these immortalized cells is not fully elucidated. To use the meibomian gland epithelial cell line as a model to investigate effects on physiological maturation of meibocytes, maturation in culture must be characterized further.

The most obvious sign for maturation of meibocytes is the accumulation of lipid droplets in the cytoplasm. Sullivan and coworkers have published the first data describing the treatment and induction of differentiation in HMGEC. According to their findings, the cells cease proliferation and differentiate under serum-containing medium [2, 3], increasing lipid accumulation in the cytoplasm of the cells [4]. Subjecting cells to azithromycin lipid storage especially in lysosomes [5, 6]. However, further experiments are needed to determine the differentiation status of these cells.

During normal maturation, the morphology of meibocytes changes from small polygonal cells to enlarged, spherical cell bodies accompanied with ultra-structural alterations and changes in protein expression. The cytoskeleton of epithelial cells is composed of various cytokeratins (CK) that can be used as biomarkers to identify epithelial subtypes and differentiation status [7]. Previous investigations showed CK6 and CK14 as markers for epithelial cells of meibomian gland ducts whereas meibomian gland azini lack CK6 and CK14 expression [8–10]. CK1 was detected in epidermal cells and the orifices of meibomian glands [11]. CK5 is a pan-epithelial marker that is expressed by meibomian gland acini, ducts, orifice, conjunctival and epidermal cells [8].

The aim of this study was to characterize meibomian gland epithelial cell differentiation in respect to ultra-structural morphology, lipid accumulation and cytokeratin expression when cells were treated with serum-free or serum-containing medium. We hypothesized that exposing immortalized HMGEC to serum would result in structural changes, production of lipid droplets and a distinct CK expression profile when compared to proliferating, serum-free cells. Morphological and cell adhesion changes can be accompanied by cell impedance variations. We therefore examined whether the medium switch caused impedance changes in HMGEC. Furthermore, we analyzed the lipid composition of the cell line. Meibocytes are specialized cells that require a sufficient supply of lipid material to fulfill their physiological task of meibum production. Therefore, culture media composition is crucial. To enhance lipid accumulation in cells we tested medium that is normally used for the cultivation of sebocytes or supplemented serum-containing medium with various components.

## Materials and Methods

### Culture of human meibomian gland epithelial cells

Experiments were conducted using the human meibomian gland epithelial cell line (HMGEC) that we received as a kind gift by David Sullivan (Schepens Eye Research Institute) and was first described in 2010 [2]. HMGEC were grown in serum-free medium (keratinocyte serum-free medium containing 5 ng/ml epidermal growth factor and 50μg/ml bovine pituitary extract). When cells reached 80–90% confluence differentiation was induced by switching to serum-containing medium (Dulbecco´s modified Eagle´s medium and Ham´s F12 containing 10% fetal calf serum (FCS) and 10 ng/ml epidermal growth factor) for 1, 3, 7, 14 or 21 days. Culture media and supplements were purchased from Gibco Life Technologies, Karlsruhe, Germany, and Biochrom AG, Berlin, Germany. To stimulate the cells, serum-containing medium was supplemented with 20% FCS, 4500 μg/ml glucose (Carl Roth, Karlsruhe, Germany), or lipid cocktail (Cat. No. 11905–031, Gibco Life Technologies, Karlsruhe, Germany), or 100μM eicosapentaenoic acid (EPA; Sigma-Aldrich, Taufkirchen, Germany), or cells were cultured in Sebomed medium (Biochrom AG, Berlin, Germany) for 1 day or 7 days.

### Sudan III lipid staining

Cells were seeded onto cover slips and fixed with 4% paraformaldehyde for 10 min at room temperature. Nuclei were stained with hemalum for 7 min and washed for 30 min. After incubation of slides in 50% ethanol for 15 sec, slides were stained with Sudan III (Merck Millipore, Darmstadt, Germany, 0.3 mg Sudan III in 70% Ethanol) for 15 min. To remove excessive stain slides were washed with 50% ethanol and then mounted on slides with Aquatex (Merck Millipore, Darmstadt, Germany). Lipid accumulation was observed under a Keyence BZ-9000 light microscope.

### Lipid quantification

To quantify the lipid droplets relative to the background image, custom image processing software was developed using MATLAB (Version R2012b, The MathWorks Inc., USA). Features corresponding to the lipid droplets were extracted by applying a custom written algorithm. Exemplary images of the processing steps are provided in S1 Fig. The image using RGB (red, green, blue) color space was transformed into a HSV (*hue*, *saturation*, *value*) color space. The HSV color space is widely used in the field of image analysis. The chromatic components hue, saturation and value correspond closely with the categories of human color perception.[12] HSV has a cylindrical geometry, with *hue* measured in degrees (0°- 359°). First, artefacts were detected and eliminated from the image by thresholding and extracting the yellow and dark blue color, which was found between 35°- 70° (hue) and 130°- 230° (hue), respectively (S1 Fig B). Second, the color range of the lipid droplets was found to be between 0° -35° and 270° -359° (*hue*), which ranges from red to purple. After this color range level was determined, everything outside this range was eliminated and the image was binarised (S1 Fig C). The white areas of the image represented the background image and black areas represented the lipid droplets (S1 Fig D). The stained area was calculated by dividing the number of black pixels (representing the lipid droplets) by the total amount of pixels of the image.

### Transmission electron microscopy

Cells grown in petri dishes were fixed in Ito's fixative (2.5% glutaraldehyde, 2.5% paraformaldehyde and 0.3% picric acid dissolved in PBS (pH 7.3) and embedded in Epon. Semi-thin sagittal sections of 1 μm were cut with a microtome (Ultracut E; Reichert Jung, Vienna, Austria) and subsequently stained with toluidine blue. Sections were viewed with an epifluorescence microscope (Aristoplan; Ernst Leitz, Wetzlar, Germany) and photographed (DC 500 camera; Leica Microsystems, Wetzlar, Germany). Ultrathin sections were stained with uranyl acetate and lead citrate and analyzed with a transmission electron microscope (EM109; Carl Zeiss Meditec GmbH, Oberkochen, Germany).

### Lipid extraction

Cells cultured for 1 or 3 days in serum-containing medium were harvested with trypsin/EDTA solution (Sigma-Aldrich, Taufkirchen, Germany) and washed three times with isotonic ammonium acetate (Merck Millipore, Darmstadt, Germany) and the cell pellet was stored in glass tubes at -80°C until extraction. Lipid extraction was performed using a modification of the method of Tran et al. [13]. Cell pellets were resuspended in 50 μL of methanol (MeOH) containing 0.01% butylated hydroxytoluene (BHT). A solvent mix of 770 μL methyl-tert butyl ether (MTBE) and 180 μL MeOH with 0.01% BHT containing internal standards was prepared. Cells were transferred to 2 mL tough tubes (Geneworks, Hindmarsh, SA, Australia), and a 500 μL aliquot of the solvent mix was used to wash the cell pellet tube and added to the tough tube. Samples were homogenized using a bead homogenizer (FastPrep-24, MP Biomedical, Seven Hills, NSW, Australia) at 6 m/s for 40 sec and the homogenate removed to 1.5 mL microcentrifuge tubes (Eppendorf, North Ryde, NSW, Australia). Homogenizing tubes were washed with 450 μL of the solvent mix and the wash added to the homogenate. Samples were vortexed for 1 hr at 4°C, and 200 μL of 150 mM ammonium acetate (LC-MS grade, Fluka, Castle Hill, NSW, Australia) was added to induce phase separation. Tubes were vortexed for 15 min and spun at 2000 g for 5 minutes to complete phase separation. The upper organic layer was removed to a new 2 mL glass vial and stored at -80°C until analysis. Extracts were diluted into methanol:chloroform (2:1 v/v) containing 5 mM ammonium acetate prior to mass spectrometric analysis.

### Mass Spectrometry

Mass spectrometric analysis was performed using a modification of the method of Brown et al. [14]. Mass spectra were acquired using a chip based nano-electrospray ionization source (TriVersa Nanomate, Advion, Ithaca, NY, USA) coupled to a hybrid linear ion trap-triple quadrupole mass spectrometer (QTRAP 5500, ABSCIEX, Foster City, CA, USA). Data were analyzed with LipidView (ABSCIEX) software version 1.1 as per the methods of Brown et al. and Tran et al. Precursor ion and neutral loss scans covering the most abundant lipid classes found in meibum (wax ester (WE), cholesterol ester (CE)) as well as abundant lipid classes in tissue (phosphatidylethanolamine (PE), phosphatidylserine (PS), phosphatidylcholine (PC), sphingomyelin (SM), ceramide (Cer), free cholesterol (Chol), diacylglycerol (DAG) and triacylglycerol (TAG)) were utilized to give coverage of both meibum and cellular lipids. MS conditions are provided in S3 Table.

### Impedance Sensing

Impedance measurements were performed with the electric cell-substrate impedance sensing (ECIS) instrument Z theta +16 (Applied Biophysics, New York, USA) using ECIS cultureware 8W10E+ arrays. Array slides were incubated with serum-free medium over night before cells were seeded at a density of 75000 cells per well. For the first 48 hours of the measurement cells were allowed to attach and grow. Then cells were either cultured in serum-free or serum-containing medium. Impedance was recorded for another 72 hours. Medium was replaced every day. Values were collected every 202 sec at 4,000 Hz as recommended by manufacture. Data were collected from 2 wells and each well had 40 electrodes. Thus each data point represents the mean ± SEM from a total of 80 electrodes. All experiments were repeated at least three times.

### RNA Preparation and cDNA Synthesis

Total RNA was isolated from cells using a TRIfast reagent (Peqlab) as described previously [15]. Contaminating DNA was digested with RNase-free DNase I (Fermentas, St. Leon-Rot, Germany) for 30 minutes at 37°C. DNase was inactivated by heating for 10 minutes at 65°C. Reverse transcription of RNA samples was performed by RevertAid H Minus First Strand cDNA Synthesis Kit (Fermentas, St. Leon-Rot, Germany) according to the manufacturer’s protocol. For each reaction, 2 μg of total RNA and 10 pmol of oligo (dT) 18 primer (Fermentas, St. Leon-Rot, Germany) were used.

### Realtime polymerase chain reaction

Realtime PCR was used to quantify the expression of desmoplakin. The selected desmoplakin primers identify transcript variant 1 (NM_004415.2) as well as transcript variant 2 (NM_001008844.1). Intron-spanning gene-specific primers were as follows: 5’-cca gaa ctc gga cgg cta c-3’ and 5’-atc aag cag tcg gag cag tt-3’ for desmoplakin and 5’-AGG GGA GAG CGG GTA AGA GA-3’ and 5’-GGA CAG GAC TAG GCG GAA CA-3’ for reference gene small ribosomal subunit (18S rRNA). Each reaction was performed in a final volume of 20 μl containing 10 μl SYBR Green mastermix (LightCycler 480 SYBR Green I; Roche, Mannheim, Germany), 0.5 μl gene-specific primer mix (10 pmol), 7.5 μl nuclease-free water and 2 μl sample cDNA. Each plate was run at 95°C for 2 min, followed by 45 cycles of 95°C for 10 s, 60°C for 10 s, and 72°C for 10 s followed by a melting curve profile (60 to 95°C) to confirm amplification of gene-specific transcript. The increase of gene-specific transcript was quantified by measuring the fluorescence of SYBR Green after each step of elongation. A standard curve was generated by five-fold serial dilutions of cDNA from human meibomian epithelial cells to calculate efficiency of realtime PCR assays. The cycle threshold (Ct) parameter is defined by 2^nd^ derivative maximum analysis with LightCyler480 software v1.5. To standardize mRNA concentration, transcript levels of the reference gene 18S rRNA were determined in parallel for each sample and relative transcript levels were corrected by normalization based on the 18S Ct levels. All realtime PCRs were performed in duplicate and the changes in gene expression were calculated using the delta delta Ct method [16]. Realtime PCR was performed using LightCycler 480 from Roche, Mannheim, Germany.

### Protein extraction

After RNA-isolation proteins were extracted from TRIfast residues. DNA was removed by ethanol precipitation and the remaining proteins were precipitated with isopropanol. Washing with guanidine hydrochloride (0,3M in 95% EtOH) was followed by dissolving the protein pellet in 10M urea/ 50mM dithiothreitol by sonication. Protein concentration of the collected supernatant was determined by Bradford assay.

### Western blot analysis

Protein containing supernatants were mixed with a reducing buffer containing β-mercaptoethanol and boiled for 5 min. Afterwards 20 μg total protein was loaded onto 12% SDS polyacrylamide gels, separated by electrophoresis and transferred to nitrocellulose membranes by electroblotting. The blot was blocked in 5% nonfat milk/TBST (1ml Tween 20 / 1 l TBS) or in 5% BSA (bovine serum albumin, Carl Roth, Karlsruhe, Germany) /TBST for 30 min and probed with primary antibody (4°C overnight, Table 1). Washing was followed by incubation with horseradish peroxidase conjugated secondary antibody (dilution 1:5000, 2h at RT). Photoreaction was determined with ECL substrate (Millipore, Schwalbach, Germany).

**Table 1:** Primary antibodies used for Western blot analysis.

Antibody	Size of detected antigen (kDa)	dilution	Catalog no.	Company
CK1	54	1:50	NCL-CK1	Novocastra
CK5	62	1:200	NB110-56916	Novus Biologicals
CK6	60	1:3000	ab93279	Abcam
CK14	50	1:150	LL002-L-CE	Novocastra
GAPDH	37	1:4000	sc-51905	Santa Cruz

### Statistics

Data are expressed as mean ± SEM. All variables were normally distributed according to Kolmogorov–Smirnov test. After evaluating values for normal distribution we performed one-way ANOVA statistics. For the interpretation of the results we used either Bonferroni or Games- Howell post hoc test depending on Levene’s test for homogeneity of variances. All data were analyzed by IBM SPSS Statistics software package version 20.0 (IBM SPSS). A *p-*value less than 0.05 was considered statistically significant.

## Results

### Time course of lipid accumulation in HMGECs

**Fig 1:**
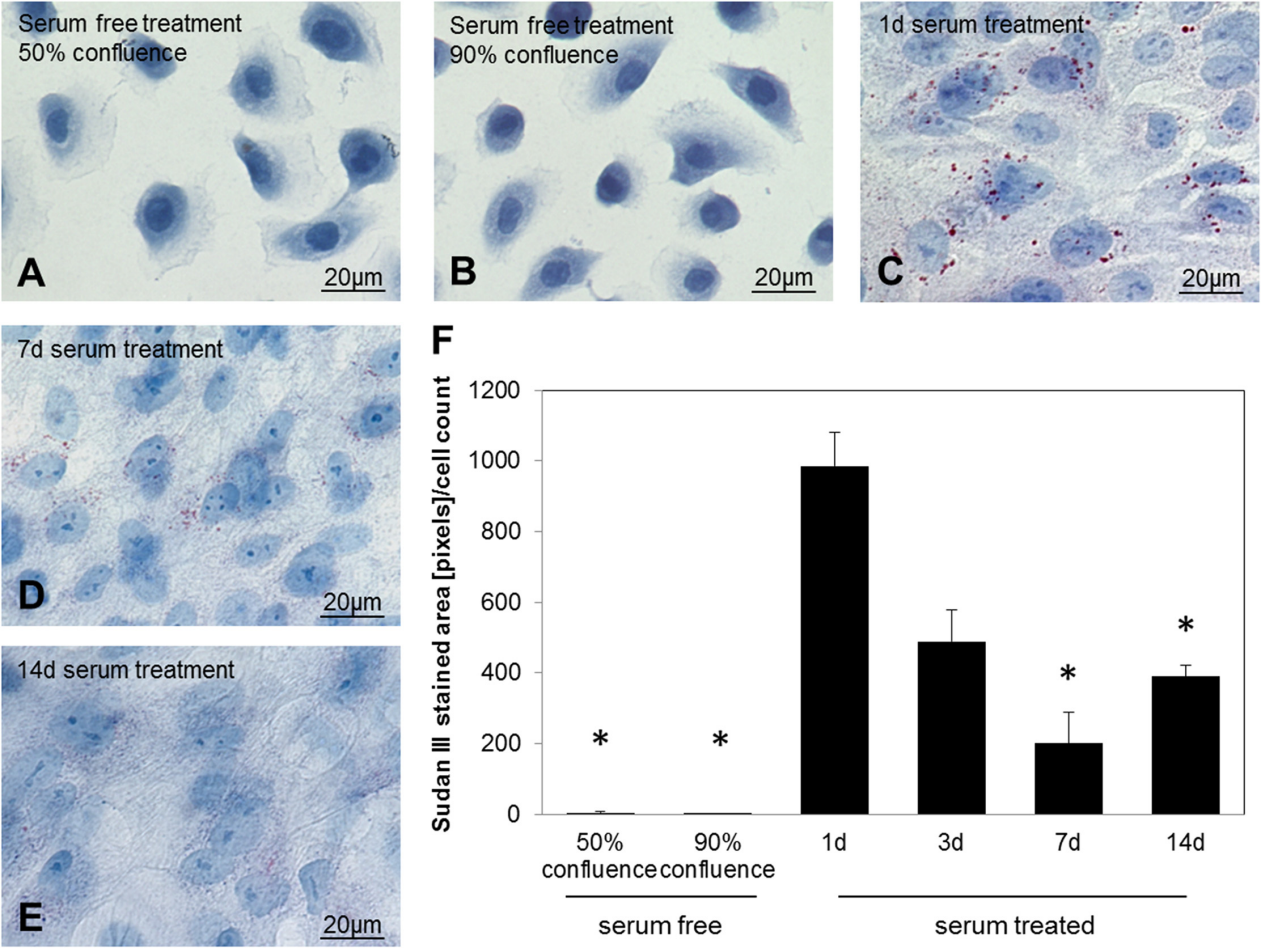
Sudan III staining for lipid detection in HMGEC. Cells were either cultured in serum-free medium until they reached 70–90% confluence (A, B) or cultured in serum-containing medium for 1 (C), 7 (D) or 14 (E) days. The graph in (F) shows quantification of Sudan III stained areas normalized to the cell count per image. Serum-free treated cells showed no lipid accumulation. Cells in serum-containing media accumulated lipids in the cytoplasm after one day treatment. Lipid accumulation decreased over time. The red stain in the pictures indicates lipid droplets.

HMGECs that were seeded and grown in serum-free medium, showed sporadic lipid accumulation at 50% and 90% confluence (Fig 1A, 1B and 1F). Cells were rounded with drop-shaped protuberances. When the medium was changed to serum-containing medium at 90% confluence, cells elongated and round gaps were formed in the cell layer that were closed after 7 days. Lipid droplets were visible after 24 hours treatment with serum-containing medium, mainly around the nuclei (Fig 1C). Quantification of Sudan III stained areas showed 985 pixels per cell (Fig 1F). The majority of cells showed lipid accumulation, however in about 20% of cells no staining was visible. After 24 hours there was a progressive decrease of lipid droplets as observed by Sudan III staining (compare Fig 1C, 1D, 1E and 1F). After 14 days lipid accumulation decreased to 380 pixels per cell (Fig 1F). Serum cultivation on glass cover slips for longer than 14 days was not possible as cells detached from the cover slips during this longer incubation period.

### Ultrastructural analysis of HMGECs under serum

**Fig 2:**
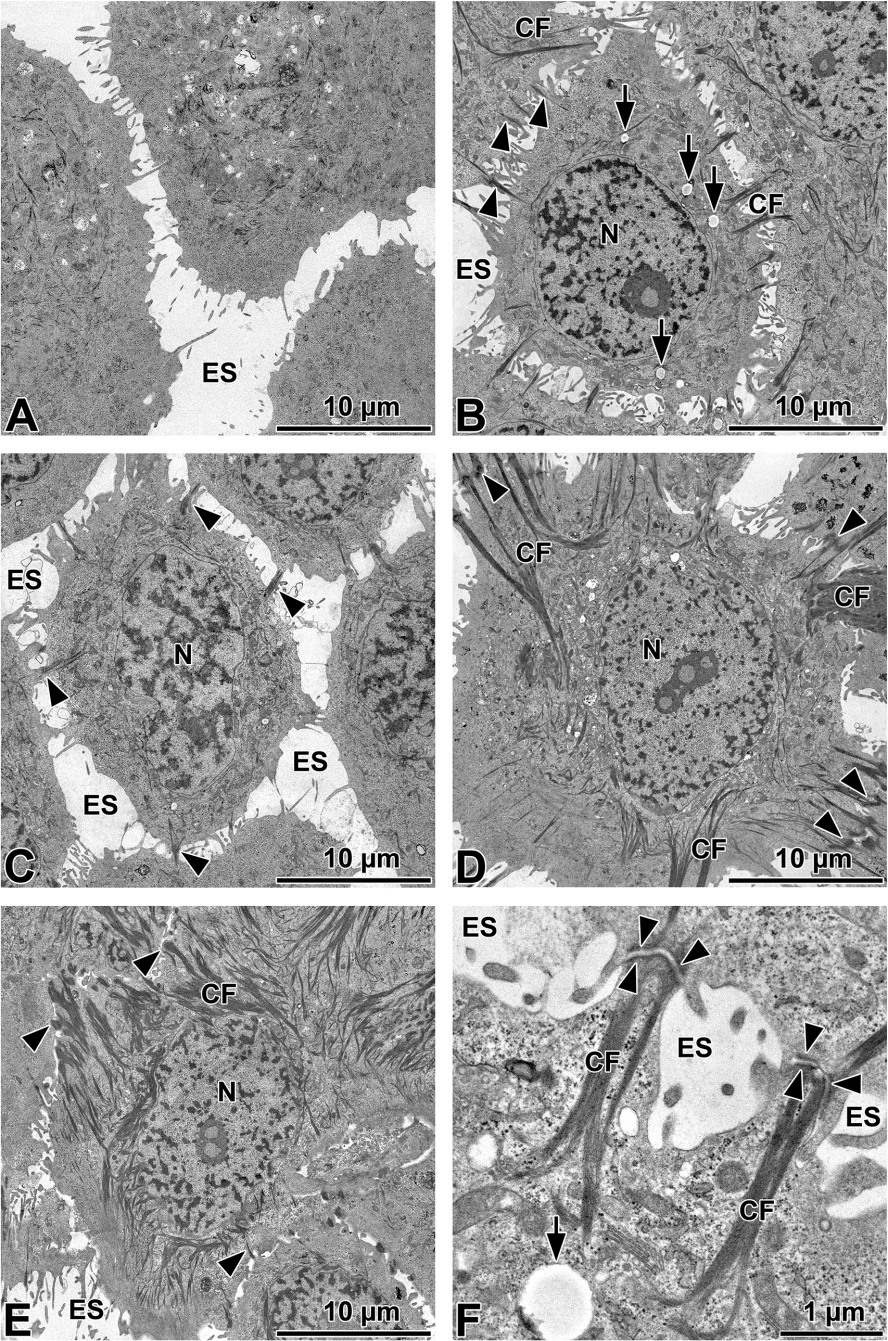
Ultra-structural analysis of HMGEC. Cells were either cultured in serum-free medium until they reached 90% confluence (A) or cultured in serum-containing medium for 1 (B, higher magnification in F), 3 (C), 7 (D) or 14 (E) days. Cytokeratin filaments (CF) elongated and desmosomes (black arrow heads) increased in serum-treated cells over time, but desmosomes were not visible in cells grown in serum-free media. Lipid droplets (arrows) can be seen in serum-treated cells. N = nuclei, ES = extracellular space.

**Fig 3:**
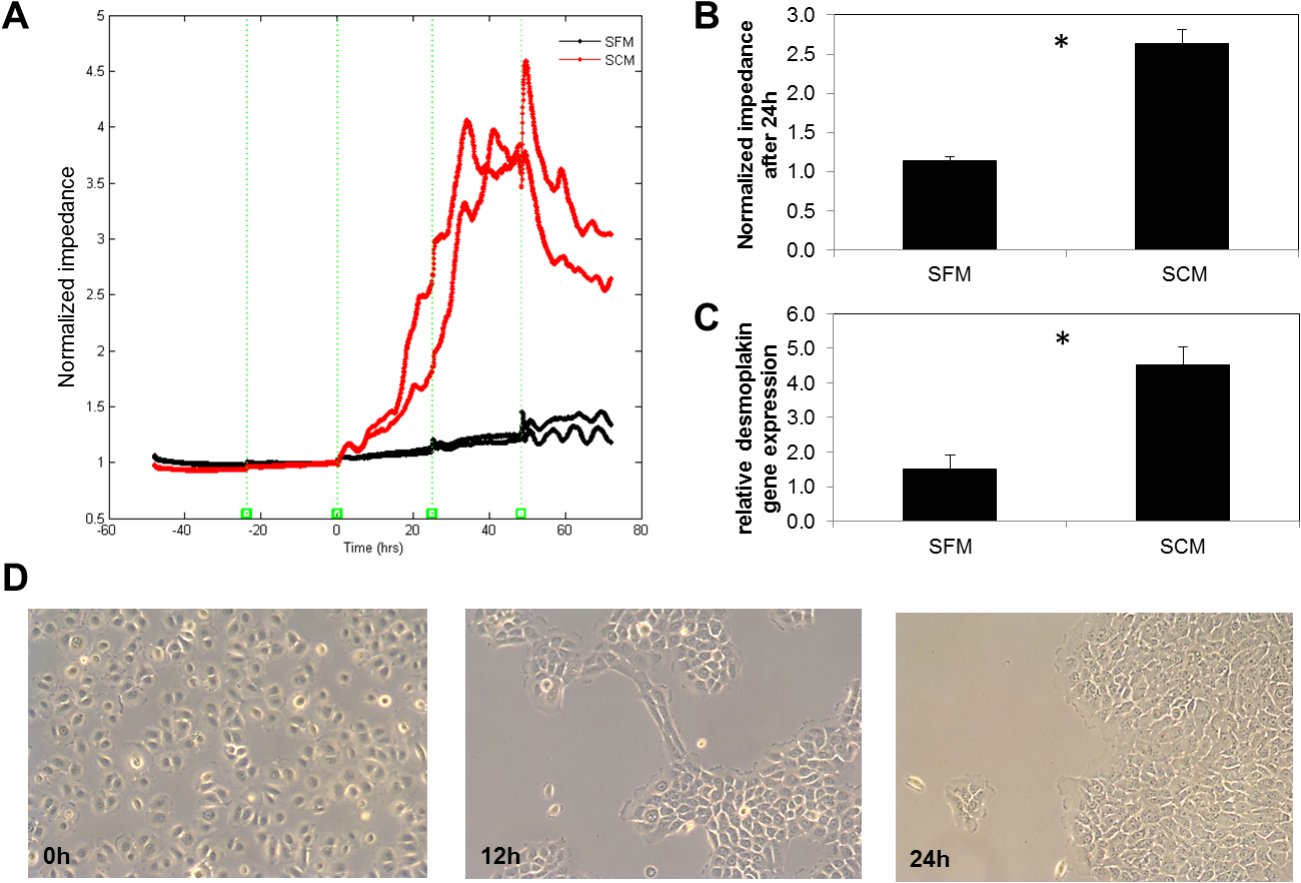
**A.** Electric Cell-substrate Impedance Sensing (ECIS) of HMGEC over 120 hours. Cells were grown in serum-free medium for 48 hours before incubation for another 72 hours in serum-free medium (SFM, black) or serum-containing medium (SCM, red). **B.** There was significantly increased impedance after 1d culture with SCM compared to SFM (* *p* ≤ 0.05, student´s t-test, n = 4). **C.** Real-time RT-PCR analyses of desmoplakin 1/2 mRNA expression in HMGEC. Cells were grown to 90% confluence followed by either cultivation in serum-free or serum-containing medium for 1 day. The fold increase transcript levels are shown as mean ± SEM and statistical significance vs. control is indicated by asterisks (n = 4, student´s t-test; * *p <* 0.05). **D.** After switching to SCM cellular morphology was changed. Previously distributed cells formed groups and cell contacts. Magnification: 10x.

Serum-free cultured HMGECs of 50% and 90% confluence displayed pseudopodia and cell processes without the formation of cell-cell-contacts (Fig 2A). Within the cells membranous lamellar inclusion bodies and some short cytokeratin filaments were visible, but no accumulation of glycogen or lipid droplets. Upon serum treatment the cell morphology changed from a rounded shape as seen in Fig 2A to an elongated shape as seen in Fig 2B (see also Fig 3D). Serum-treatment for 1 day induced formation of long cytokeratin filaments and desmosomes which increased in number over time (Fig 2B, 2C, 2D, 2E and 2F). After 7 and 14 days culture in serum-containing medium large cytokeratin accumulations were observed (Fig 2D and 2E). The amount of glycogen and membranous lamellar inclusion bodies varied within the cytoplasm of differentiated cells. Lipid vesicles that were not electron dense were visible after 1 day of serum cultivation but vesicle number decreased over time. Electron dense lysosomes were not visible. Additional images with descriptions are provided in S2 Fig.

### Impact of serum on cell impedance and desmosome formation in HMGECs

Cell impedance was assessed using ECIS for 120 hours. Fig 3A shows the time course of the normalized impedances of 4 wells with 40 electrodes each. A seeding cell number of 75000 cells/well generated a confluent cell layer after 24 hours according to preliminary studies. Cells seeded in serum-free medium were allowed to attach and grow for 48 hours. However, only a minimal increase in impedance advance was observed and the expected plateau did not occur. This indicated that cells were confluent but did not form desmosomes. After 48 hours preincubation in serum-free medium, cells were either retained in serum-free medium or cultured in serum-containing medium. Serum-free treated cells showed no significant increase of the impedance in the following 72 hours. In contrast the serum-cultured cells showed a 2.3-fold elevation in impedance after 24 hours (Fig 3B). The elevated impedance was related to an intense change in cell morphology and cell contact upon serum treatment (Fig 3D). After 1d stimulation of HMGEC with serum-containing medium desmoplakin 1/2 gene expression was elevated 3-fold compared to cells that were not exposed to serum (Fig 3C).

### Cytokeratin expression in HMGECs

**Fig 4:**
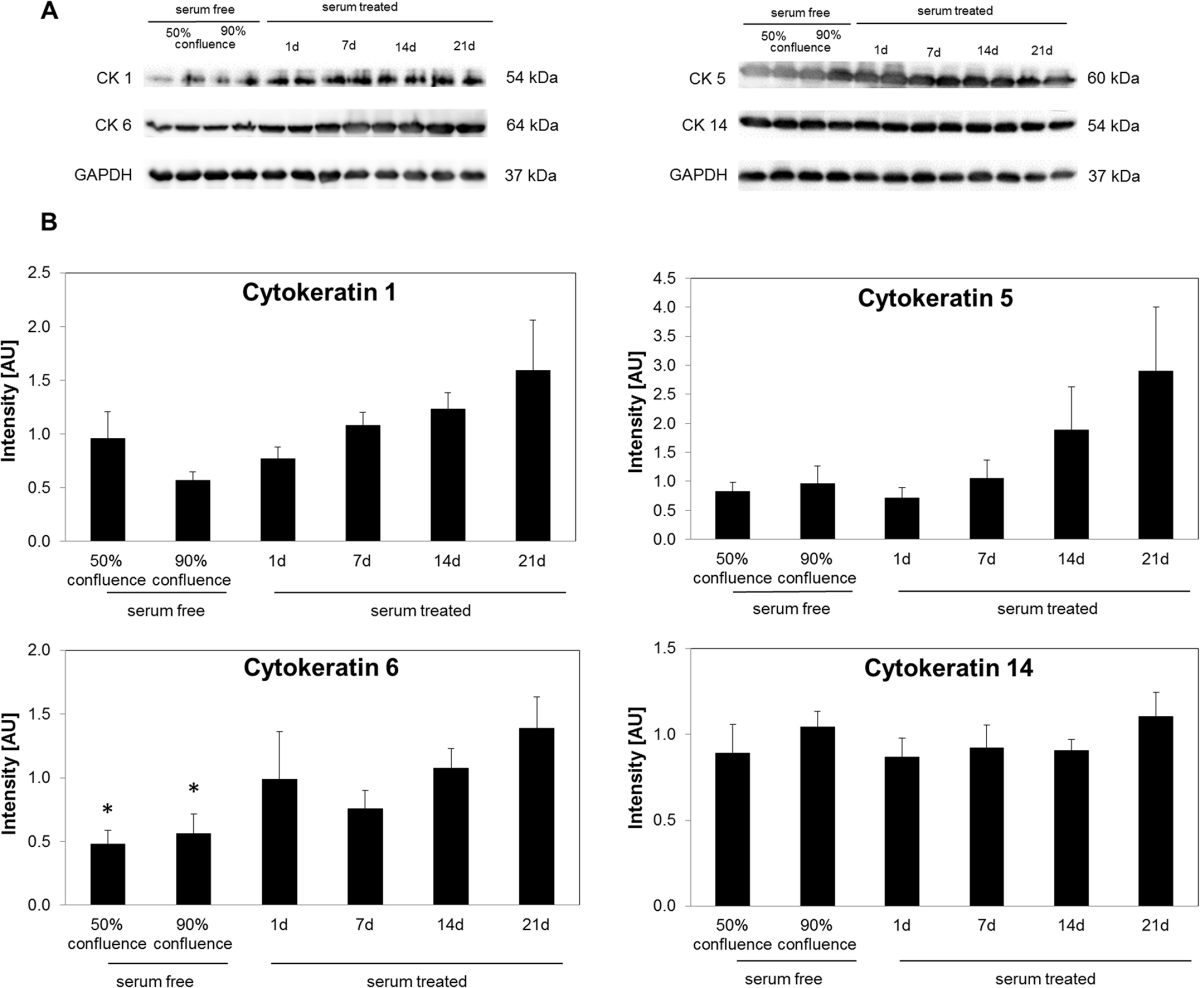
Expression of cytokeratins (CK) and GAPDH in HMGEC. (A) Representative western blots show specific bands for GAPDH, CK1, CK5, CK6 and CK14. (B) Intensities (normalized to GAPDH) are shown as mean ± SEM and statistical significance vs. 21d serum treatment is indicated by asterisks (n = 6, one-way ANOVA; * *p <* 0.05).

The expression profiles of CK1, 5, 6 and 14 in HMGECs at different time points are shown in Fig 4. CK 1 and 5 expression profiles followed an exponential shape. The expression levels of CK1 and CK5 in cells treated for 21 days with serum were unchanged when compared to serum-free cultured cells. CK6 protein expression increased over time with the most intense bands being seen after 21 days cultivation in serum-containing medium. Cells that were cultured for 21 days in serum containing medium showed a significant 2.9-fold (p = 0.017) and 2.5- fold (p = 0.025) elevation of CK6 expression compared to 50% or 90% confluent serum-free treated cells respectively. CK14 protein expression was not significantly altered under different cultivation conditions.

### Lipid profile of serum-treated HMGECs

**Fig 5:**
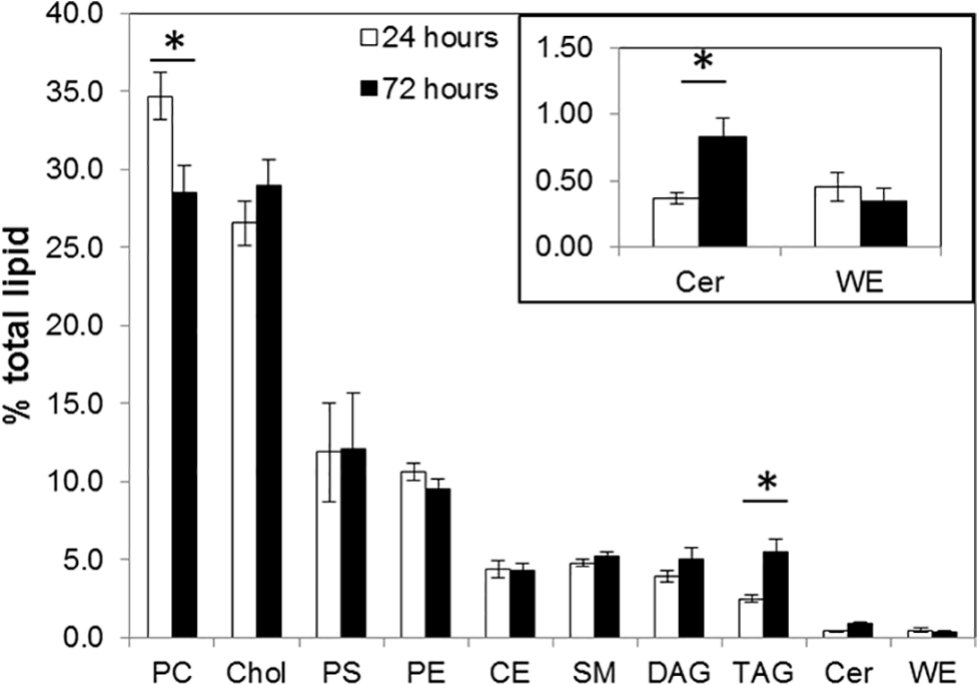
Quantitative analysis of lipid profile of HMGEC cultivated for 1 day or 3 days in serum-containing medium. Values are shown as the mean of 12 measurements ± SEM (n = 15). Phosphatidylcholine (PC), cholesterol (Chol), phosphatidylserine (PS), phosphatidylethanolamine (PE), cholesterol ester (CE), sphingomyelin (SM), diacylglycerol (DAG), triacylglycerol (TAG), ceramide (Cer) and wax ester (WE) were detectable. (Student´s t-test; * *p ≤* 0.001)

The lipid spectrum of HMGECs is shown in Fig 5. After 1 day or 3 days of serum-culture the HMGEC lipidome was dominated by phospholipids (PC + PS +PE + SM; >50%) and free cholesterol (26–28%). WE and CE are the dominant lipid classes found in meibum [14, 17] [18], however they were found to be in relatively low concentrations in the extracts from HMGEC cells, with CE at approximately 5% and WE less than 1% of total lipid in the serum-treated HMGEC cells. Moreover, (O-acyl)-omegahydroxy fatty acids (OAHFAs) found exclusively in meibum where not detected in the lipid extracts. Only a small difference in lipid profile was observed after 3 days of serum treatment compared to 1 day of treatment. PC levels were reduced by 82% (*p =* 0.0014), whereas TAG and Cer levels were elevated (*p ≤* 0.001; 220% and 224% respectively). All lipid species identified are listed in S3 Table (CE), S4 Table (phospholipids), S5 Table (DAG and TAG), and S6 Table (WE).

### Effect of 20% FCS, EPA, lipid cocktail, high glucose and Sebomed medium on HMGEC lipid accumulation and ultra-structure

**Fig 6:**
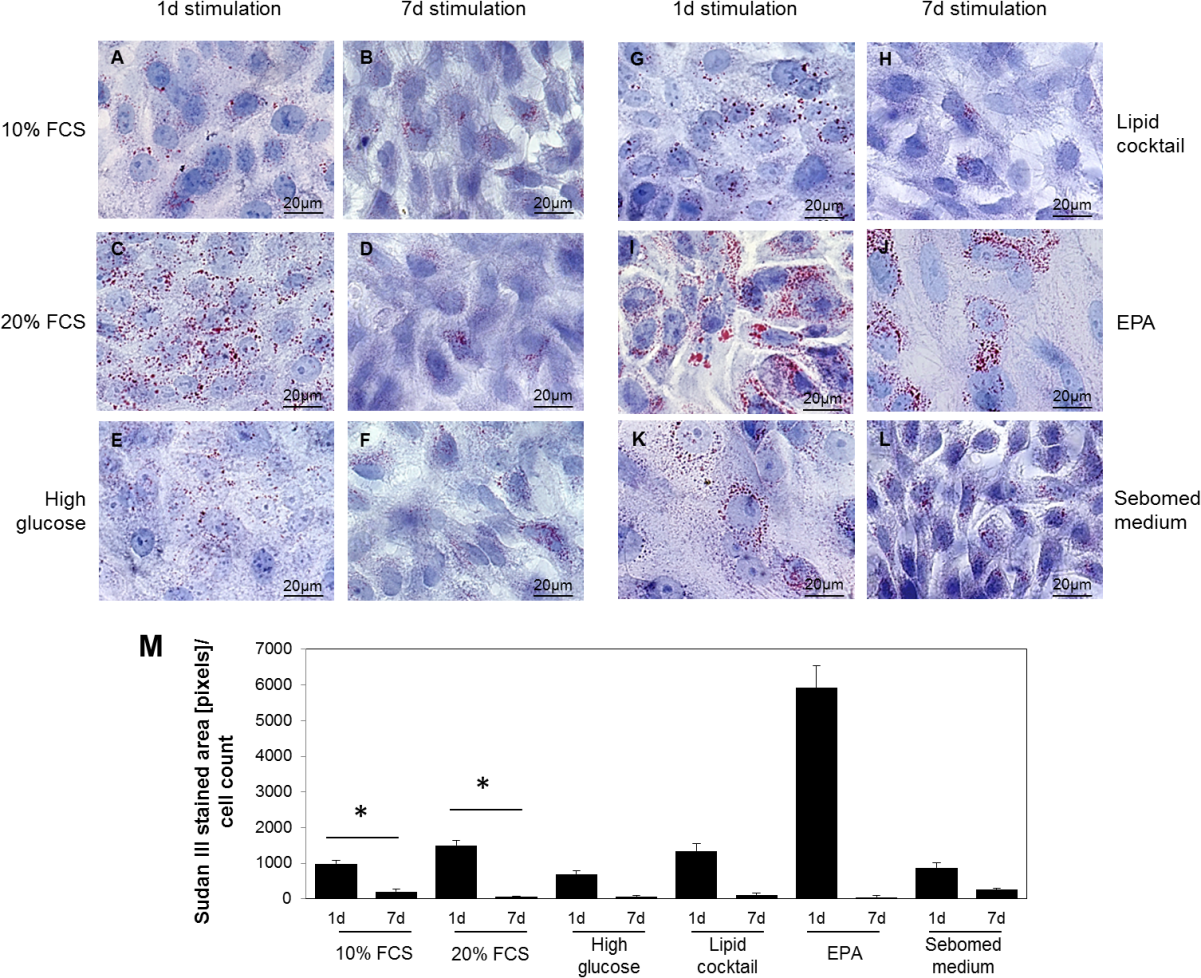
A. Sudan III staining to visualize lipid accumulation of HMGEC after 1 day or 7 days stimulation with addition of 10% FCS (C, D), high glucose (E, F), lipid cocktail (G, H), 100μM EPA (I, J) in10% serum-containing medium (A, B) or sebomed medium (K, L). Fig 6M shows quantification of Sudan III stained areas normalized to the cell count per image. In general, lipid accumulations were more prominent after 1 day compared to 7 days cultivation in serum-containing medium. Highest levels of lipids were visible after 1 day treatment with 100μM EPA (I). Red stain indicates lipid droplets.

Serum-containing medium was supplemented with either an additional 10% FCS (to 20% total) (Fig 6C and 6D), high glucose (Fig 6E and 6F), a lipid cocktail (Fig 6G and 6H), 100μM EPA (Fig 6I and 6J), or replaced by sebomed medium (Fig 6K and 6L) for 1 or 7 days. In general, lipid droplets were more prominent and numerous after 1 day than 7 days of treatment (Fig 6M). The most abundant lipid droplets were visible in cells cultured with EPA (Fig 6I and 6M; 5900 pixels per cell). There was no difference in lipid droplet numbers noticeable between controls (10% FCS) and cells supplemented with higher FCS levels, lipid cocktail or high glucose in serum-containing medium or sebomed medium.

**Fig 7:**
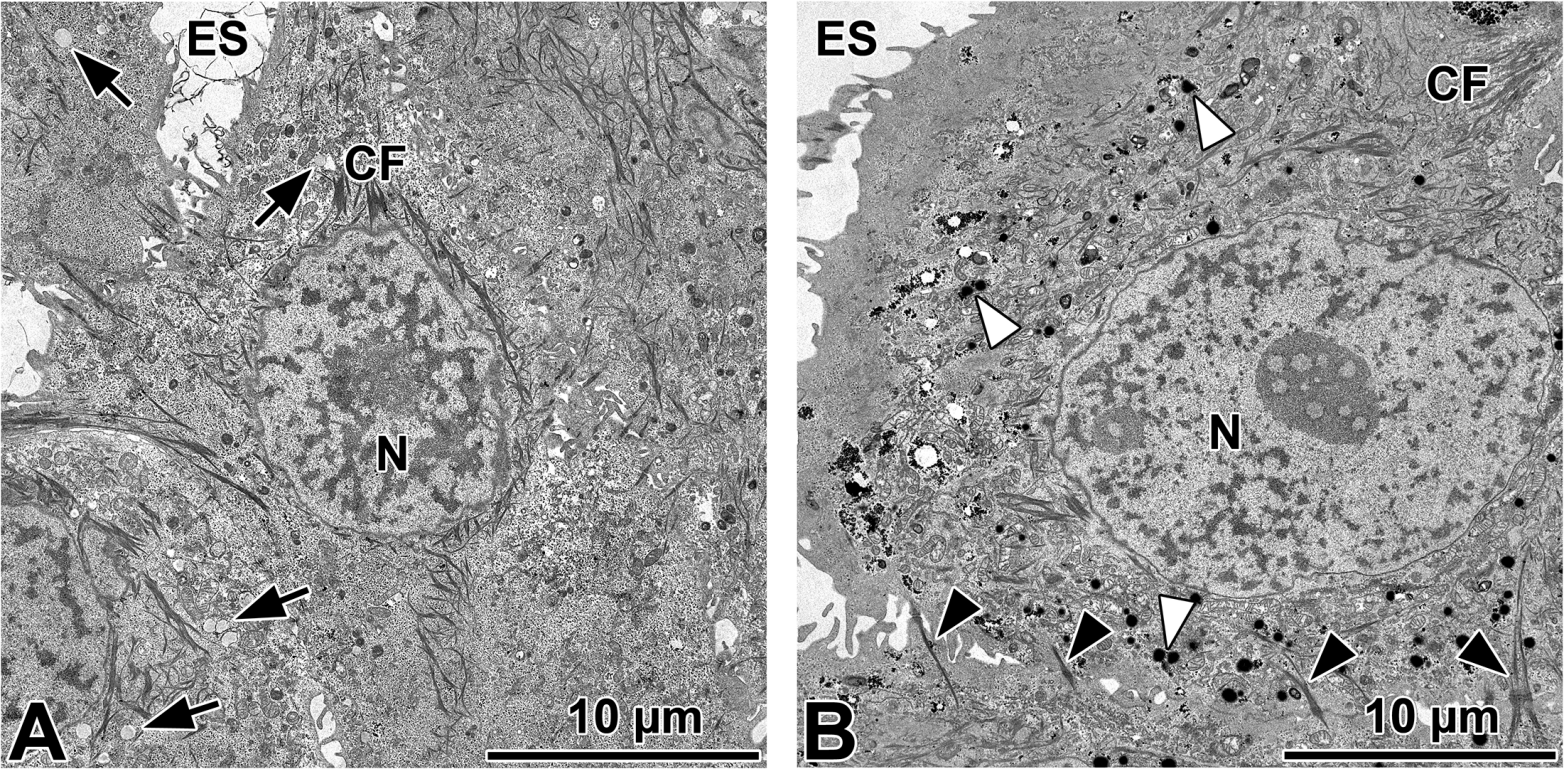
Ultra-structural analysis of HMGEC after 1 day stimulation with serum-containing medium supplemented with 100 μM EPA (A) and 20% FCS (B). Cytokeratin filaments (CF), desmosomes (black arrow heads) and lipid droplets (arrows) are visible in 20% FCS treated cells. EPA stimulated cells show numerous lysosomes (white arrow heads).

Subsequent ultra-structural analysis of cells treated with high FCS or EPA for 1 day showed cytokeratin filaments and desmosome formation (Fig 7). Cells treated with high FCS showed low-contrast vesicles that resemble fat vesicles (Fig 7A). EPA stimulated cells contained numerous electron dense lysosomes (Fig 7B).

## Discussion

Meibomian gland dysfunction (MGD) is the main cause for DED and is accompanied by a loss and atrophy of meibum producing cells, hyperkeratinization of the meibomian gland ducts, and an increase in meibum viscosity [19]. The meibomian gland epithelial cell line (HMGEC), first described in 2010 [2], may be able to serve as a model to study the physiology and pathophysiology of MGD *in vitro*.

According to the culture protocol established by Sullivan and coworkers, HMGECs differentiate under serum treatment [2–4]. This means that cells halt proliferation, accumulate lipids and regulate genes associated with lipogenesis [2, 3]. We used the published protocol for the cultivation of HMGEC and examined lipid accumulation in cells cultured in the presence of serum for up to 14 days. Serum-free cultured HMGECs showed only a few lipid droplets. After 24 hours of serum treatment lipid droplets were visible but their number decreased from day 3 onwards. Corresponding ultrastructural analysis of HMGECs confirmed that lipid droplets were visible after serum treatment for 24 hours, and disappeared over time. Meibum vesicles increase in size and number within meibomian acinar cells from basal to differentiating and mature cells [19–21]. Furthermore, the location of lipid synthesis is the smooth endoplasmatic reticulum, as can be found in tissue sections of meibomian glands [20]. These immortalized HMGECs contain rough but not smooth endoplasmatic reticulum indicating enhanced protein rather than lipid synthesis (data not shown).

Jester et al. found that there is no transition zone between ductal epithelium and acinar cells in tissue sections of meibomian glands. Cells were either keratinizing cells with “lamellar bodies” or keratohyaline granules, or cells with lipid vesicles [20]. In the current study serum free cultured HMGECs rarely contained cytokeratin filaments. Upon serum treatment HMGECs exhibited considerable cytokeratin filaments. From day 7 onwards cytokeratin filaments formed larger aggregates. In an analysis of HMGECs Liu et al. described intraepithelial structures that they termed “lamellar bodies” [6]. However, this term is misleading, as the structures morphologically have no similarity to lamellar bodies in pneumocytes of the lung where they occur as granules that are surrounded by a membrane and contain multilamellar lipid membranes. The only similarity is lipid is stored in both types of granules. Our present investigation revealed similar vesicles under all cultivation conditions in HMGECs to those described by Liu et al. [6] as lamellar bodies. However, as pneumocytes belong to a completely different cell type than meibocytes, no lamellar bodies were visible in the granules of HMGECs and the lipid (as determined in this study), and protein (for example lamellar bodies of the lung containing surfactant proteins) content differs significantly, we prefer to term the observed granules in HMGECs simply as lipid granules.

From the morphological aspect, HMGECs cannot be classified into ductal or acinar cells because they show characteristics of both. The report of the TFOS Workshop on meibomian gland dysfunction points out that basal acinar cells might transform to ductal cells [19] which indicates that there is not a strict separation of both cell populations. This mechanism could happen to HMGECs when cells are cultured in serum containing medium.

Serum free cultured HMGECs showed cell processes without the formation of cell contacts. Surprisingly, HMGECs started to form desmosomes upon serum treatment. More and more desmosomes were visible over time. The visible formation of desmosomes was accompanied by a 3-fold elevation of desmoplakin1/2 gene expression. Desmoplakin isoforms 1 and 2 bind cytokeratin filaments to desmosomal plaques [22]. This finding is supported by the electric impedance sensing measurements where current flows over the gold electrode on the bottom of the arrays and the impedance can be determined. Cells on the electrode act as insulators and thereby increase impedance and resistance [23]. Currents take the path of least resistance so that at 4000Hz currents mainly take a route around the cells and not through cell membranes [24]. Normally, once a confluent cell layer with cell contacts is established the normalized impedance reaches a plateau. The impedance of serum-free cultured HMGECs continuously increased even after cells reached optical confluence. When cells were treated with serum containing medium the normalized impedance rose and was 2.3-fold higher after 24 hours compared to serum-free treated cells. This increase was due to the formation of desmosomes, morphological changes and narrowing of cell distances.

Visible cytokeratin filaments after serum cultivation might be due to CK1, 5 and 6 protein upregulation. Western blot analysis showed an increasing CK6 protein production upon serum treatment with highest levels after 21 days. Interestingly, CK1 is a marker for epidermal cells and the orifices of meibomian glands [11] and CK6 is a marker for epithelial cells of meibomian gland ducts [8–10]. CK5 can be detected in meibomian gland acini, ducts, orifices, conjunctival and epidermal cells [8]. CK14 was not upregulated upon serum treatment. The increasing expression of CK1 and CK6 supports the assumption that HMGECs transform to keratinizing cells when cultured in the presence of serum.

High calcium levels can induce differentiation of epidermal cells including the formation of desmosomes [25, 26]. Keratinization of HMGECs might be induced by the higher calcium levels found in the serum containing medium. The calcium concentration of Dulbecco´s modified Eagle´s medium and Ham´s F12 is approximately 100-fold higher than keratinocyte serum-free medium and its supplements. Investigations are continuing into this aspect of HMGEC differentiation.

Lipid analysis of 1 and 3 day serum cultured cells showed that the most abundant lipids were phospholipids, contributing over 50% of the total lipidome. Previously published lipid composition of HMGECs after 14 days serum treatment revealed over 70% phospholipids [27]. This difference might be due to the longer cultivation period in the previous study. Nevertheless, the lipidomes of meibum and tears contain very low levels of phospholipids [14, 17, 28–33]. The major components of meibum and tears are wax esters and cholesteryl esters [14, 34–36] that were found to be of low abundance in cultured HMGECs. Moreover, no OAHFAs were detected in HMGECs, a lipid class produced exclusively by meibomian glands [14]. These data are consistent with those reported for HMGECs previously [4] (for comparison see Table 2). Comparison of relative lipid levels of serum-treated HMGECs with the membrane lipidome of Madin–Darby canine kidney cells [37] underlines that the lipidome of HMGECs predominately derives from membranes and resembles epithelial cells (Table 2). Comparing the lipidome of the HMGECs and epidermal cells shows similar levels of cholesterol, but no free fatty acids and lower levels of sphingolipid in HMGECs [38, 39]. These findings suggest that the HMGECs do not originate from epidermal cells.

**Table 2:** Comparison of lipids reported in the current study to those reported for HMGECs [4] and epithelial cells [37] in previous reports.

	Sullivan et al (14 days) [4]		Sampaio et al (3 days) [37]		Hampel et al (3 days)		
	% lipids (in common in all 3 studies)	% lipid (Sullivan & Hampel)	% lipids (in common in all 3 studies)	% lipid (Sampaio & Hampel)	% lipids (in common in all 3 studies)	% lipid (Sullivan & Hampel)	% lipid (Sampaio & Hampel)
PE	2.6	2.4	15.2	14.8	10.8	10.3	10.1
PS	3.6	3.4	8.3	8.1	14.5	13.7	13.5
PC	37.9	35.5	34.5	33.7	33.7	32.0	31.5
SM	16.8	15.8	9.3	9.1	6.0	5.7	5.6
DAG				1.7			5.6
Cer				0.8			1.0
CE		4.4				4.6	
Chol	39.1	36.7	32.6	31.8	34.9	33.2	32.6
WE		1.9				0.5	

phosphatidylethanolamine (PE), phosphatidylserine (PS), phosphatidylcholine (PC), sphingomyelin (SM), diacylglycerol (DAG), ceramide (Cer), cholesterol ester (CE), free cholesterol (Chol), and wax ester (WE)

Our findings confirm that HMGECs can accumulate lipid, but the lipid profile was different to that expects of meibomian gland cells. Therefore, we tested different supplements that might influence lipid production by the HMGECs. Supplementation with EPA increased the number of lipid droplets after 1 day. EPA supplementation caused the formation of electron dense vesicles (Fig 7) that may be lysosomes. It has been described previously that lipids are found in lysosomes of HMGECs [6]. In ultrastructural investigations of meibomian gland sections, lysosomes were only found in differentiating acinar cells [20]. Meibomian lipid vesicles have not been associated with lysosomes but are sometimes associated with the smooth endoplasmatic reticulum [20].

The lipid accumulation in supplemented HMGECs decreased after 7 days. Under the cultivation conditions in the present study, HMGECs produced lipid vesicles to a certain degree but maturation processes halted or are even reversed this process and full maturation into meibocytes is not achieved. Instead under the various culture conditions used in this study HMGECs became keratinizing epithelial cells with similarities to the epithelial cells of the excretory ducts of meibomian glands. Therefore cultivation of the cells under the described conditions for longer than 3 days are good models to study keratinization processes. These processes are known to occur during DED in the excretory duct system of meibomian glands. Short term cultivation (24 h) seems to be useful for meibocyte differentiation studies.

In summary, HMGECs are currently the only available immortalized cell line to study the physiology of meibomian gland epithelial cells. However, *in vivo* meibomian gland epithelial cells undergo differentiation and maturation processes that can not be simulated *in vitro* by the previously published cultivation protocol [2]. The phenotype of the cells can vary under different culture conditions. Our investigations indicate that HMGECs undergo keratinization processes under serum treatment. Hyperkeratinization is one of the reasons for meibomian gland dysfunction. HMGEC line should be investigated in future studies to determine whether it can be used as a model to investigate keratinization processes and the pathophysiology of meibomian gland dysfunction.

## Supporting Information

S1 Fig Process for image analysis with the custom made algorithm; Original image (A). Detection of artefacts (B). Thresholded image of the lipid droplets (C). Overlay of extracted lipids (black) on the original image (D).
(DOCX)

S2 Fig Ultra-structural analysis of HMGEC. **A, B, C.** HMGECs were cultured in serum-free medium until they reached 90% confluence. Cells show a round shape with pseudopodia (higher magnification in B). Short cytokeratin filaments, mitochondria and membranous lamellar inclusion bodies are visible within the cytoplasm (higher magnification in C). **D, E, F.** HMGECs were cultured in serum-containing medium for 1 day. Membranous lamellar inclusion bodies and some lipid droplets can be found in serum-treated cells (higher magnification in E). Cells are seen to start to form desmosomes (higher magnification in F). **G, H, I.** HMGECs were cultured in serum-containing medium for 14 days. Cytokeratin filaments elongate and surround the nucleus and irradiate into desmosomes. Desmosomes increase in number and size in serum-treated cells over time (higher magnification in H). Glycogen accumulations and membranous lamellar inclusion bodies can be found within the cytoplasm (higher magnification in I).
(DOCX)

S1 Table Target lipid class, ion mode, MS/MS experiment (precursor ion (PI) or neutral loss (NL)), and CID energy. (DOCX)

S2 Table Lipid class means and standard error (n = 15) in HMGEC cultivated for 1 day or 3 days in serum-containing medium. All measurements are listed as mol% of total lipid.
(DOCX)

S3 Table CE molecular lipid means and standard error (n = 15) in HMGEC cultivated for 1 day or 3 days in serum-containing medium. All measurements are listed as mol% of total lipid.
(DOCX)

S4 Table PL molecular lipid means and standard error (n = 15) in HMGEC cultivated for 1 day or 3 days in serum-containing medium. All measurements are listed as mol% of total lipid.
(DOCX)

S5 Table DAG and TAG molecular lipids, mean and standard error (n = 15) in HMGEC cultivated for 1 day or 3 days in serum-containing medium. All measurements are listed as mol% of total lipid.
(DOCX)

S6 Table WE molecular lipids, mean and standard error (n = 15) in HMGEC cultivated for 1 day or 3 days in serum-containing medium. All measurements are listed as mol% of total lipid.
(DOCX)
