# Supplementary material for: Serum-Induced Keratinization Processes in an Immortalized Human Meibomian Gland Epithelial Cell Line
Source: PLoS One. 2015 Jun 4;10(6):e0128096. doi: 10.1371/journal.pone.0128096 (PMC4456149; doi:10.1371/journal.pone.0128096)
Supplement: S1 Fig — Original image (A). Detection of artefacts (B). Thresholded image of the lipid droplets (C). Overlay of extracted lipids (black) on the original image (D). (DOCX) [file pone.0128096.s001.docx]

**Serum-induced keratinization processes of human meibomian gland epithelial cells**

Ulrike Hampel; Antje Schröder; Todd Mitchell; Simon Brown; Peta Snikeris; Fabian Garreis; Carolina Kunnen; Mark Willcox; Friedrich Paulsen

**Supporting information**

| **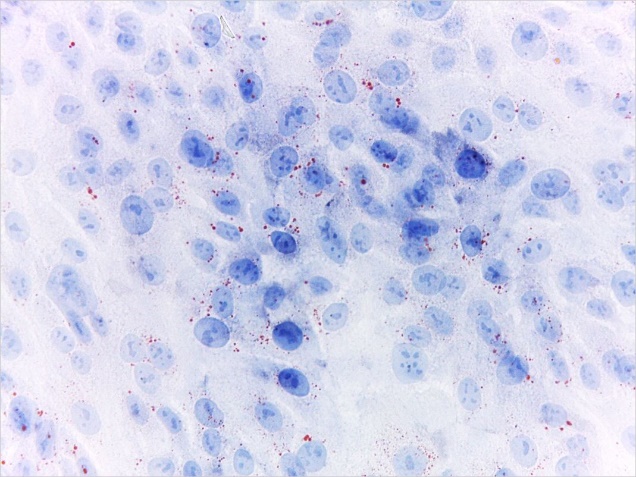**  **A** | **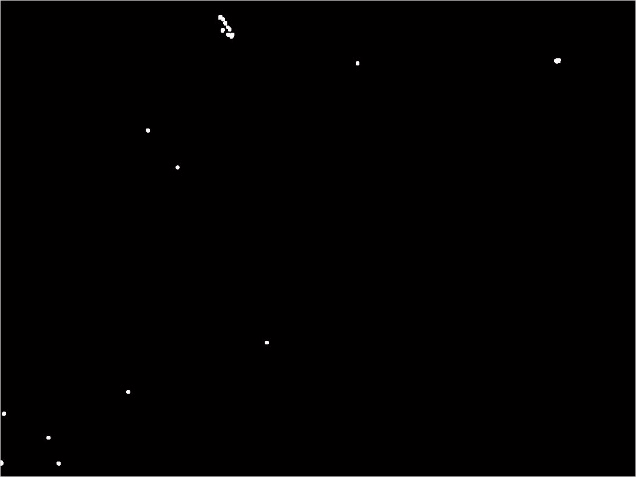**  **B** |
| --- | --- |
| **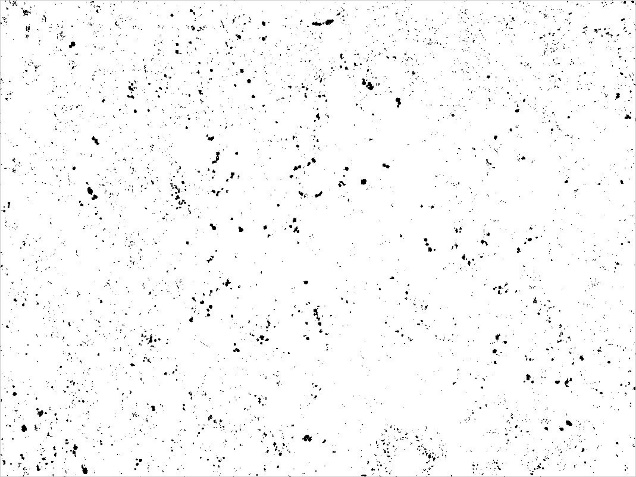**  **C** | **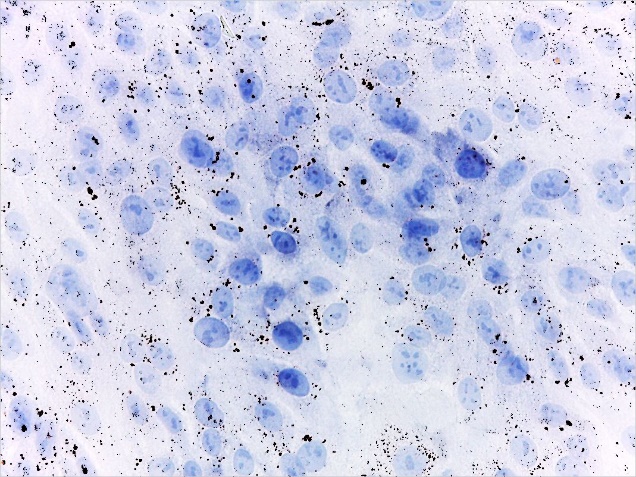**  **D** |

**S1 Figure .** Process for image analysis with the custom made algorithm; Original image (A). Detection of artefacts (B). Thresholded image of the lipid droplets (C). Overlay of extracted lipids (black) on the original image (D).
