# Supplementary material for: Serum-Induced Keratinization Processes in an Immortalized Human Meibomian Gland Epithelial Cell Line
Source: PLoS One. 2015 Jun 4;10(6):e0128096. doi: 10.1371/journal.pone.0128096 (PMC4456149; doi:10.1371/journal.pone.0128096)
Supplement: S2 Fig — A, B, C. HMGECs were cultured in serum-free medium until they reached 90% confluence. Cells show a round shape with pseudopodia (higher magnification in B). Short cytokeratin filaments, mitochondria and membranous lamellar inclusion bodies are visible within the cytoplasm (higher magnification in C). D, E, F. HMGECs were cultured in serum-containing medium for 1 day. Membranous lamellar inclusion bodies and some lipid droplets can be found in serum-treated cells (higher magnification in E). Cells are seen to start to form desmosomes (higher magnification in F). G, H, I. HMGECs were cultured in serum-containing medium for 14 days. Cytokeratin filaments elongate and surround the nucleus and irradiate into desmosomes. Desmosomes increase in number and size in serum-treated cells over time (higher magnification in H). Glycogen accumulations and membranous lamellar inclusion bodies can be found within the cytoplasm (higher magnification in I). (DOCX) [file pone.0128096.s002.docx]

**Serum-induced keratinization processes of human meibomian gland epithelial cells**

Ulrike Hampel; Antje Schröder; Todd Mitchell; Simon Brown; Peta Snikeris; Fabian Garreis; Carolina Kunnen; Mark Willcox; Friedrich Paulsen

**Supporting information**





**S2 Figure.** Ultra-structural analysis of HMGEC.

**A, B, C.** HMGECs were cultured in serum-free medium until they reached 90% confluence. Cells show a round shape with pseudopodia (higher magnification in B). Short cytokeratin filaments, mitochondria and membranous lamellar inclusion bodies are visible within the cytoplasm (higher magnification in C).

**D, E, F.** HMGECs were cultured in serum-containing medium for 1 day. Membranous lamellar inclusion bodies and some lipid droplets can be found in serum-treated cells (higher magnification in E). Cells start forming desmosomes (higher magnification in F).

**G, H, I.** HMGECs were cultured in serum-containing medium for 14 days. Cytokeratin filaments elongate and surround the nucleus and irradiate into desmosomes. Desmosomes increase in number and size in serum-treated cells over time (higher magnification in H). Glycogen accumulations and membranous lamellar inclusion bodies can be found within the cytoplasm (higher magnification in I).
