# Supplementary material for: Serum-Induced Keratinization Processes in an Immortalized Human Meibomian Gland Epithelial Cell Line
Source: PLoS One. 2015 Jun 4;10(6):e0128096. doi: 10.1371/journal.pone.0128096 (PMC4456149; doi:10.1371/journal.pone.0128096)
Supplement: S1 Table — (DOCX) [file pone.0128096.s003.docx]

**Serum-induced keratinization processes of human meibomian gland epithelial cells**

Ulrike Hampel; Antje Schröder; Todd Mitchell; Simon Brown; Peta Snikeris; Fabian Garreis; Carolina Kunnen; Mark Willcox; Friedrich Paulsen

**Supporting information**

**S1 Table.** Target lipid class, ion mode, MS/MS experiment (precursor ion (PI) or neutral loss (NL)), and CID energy.

| Target Lipid | Ion mode | MS/MS | CID energy |
| --- | --- | --- | --- |
| phosphatidylcholine (PC) | + ve | PI 184.1 *m/z* | 40 |
| lysophosphatidylcholine (LPC) | + ve | PI 184.1 *m/z* | 30 |
| sphingomyelin (SM) | + ve | PI 184.1 *m/z* | 40 |
| ceramide (Cer) | + ve | PI 264.4 *m/z* | 35 |
| phosphatidylethanolamine (PE) | + ve | NL 141 Da | 30 |
| phosphatidylserine (PS) | + ve | NL 185 Da | 25 |
| cholesterol ester (CE) | + ve | PI 369.4 *m/z* | 25 |
| free cholesterol (Chol) | + ve | PI 369.4 *m/z* | 15 |
| triacylglycerol (TAG) | | | |
| 14:0 | + ve | NL 245.2 Da | 35 |
| 16:1 | + ve | NL 271.3 Da | 35 |
| 16:0 | + ve | NL 273.3 Da | 35 |
| 18:2 | + ve | NL 297.3 Da | 35 |
| 18:1 | + ve | NL 299.3 Da | 35 |
| 18:0 | + ve | NL 301.3 Da | 35 |
| 20:4 | + ve | NL 321.3 Da | 35 |
| 22:6 | + ve | NL 345.3 Da | 35 |
| diacylglycerol (DAG) | | | |
| 16:1 | + ve | PI 311.3 *m/z* | 32 |
| 16:0 | + ve | PI 313.3 *m/z* | 32 |
| 17:0 | + ve | PI 327.3 *m/z* | 32 |
| 18:2 | + ve | PI 337.3 *m/z* | 32 |
| 18:1 | + ve | PI 339.3 *m/z* | 32 |
| wax ester (WE) |  |  |  |
| 16:1 | + ve | PI 255.2 *m/z* | 25 |
| 16:0 | + ve | PI 257.2 *m/z* | 25 |
| 17:0 | + ve | PI 271.2 *m/z* | 25 |
| 18:1 | + ve | PI 283.2 *m/z* | 25 |
| 18:0 | + ve | PI 285.2 *m/z* | 25 |
| (O-acyl)-omegahydroxy fatty acid (OAHFA) | | | |
| 16:1 | - ve | PI 253.3 *m/z* | 45 |
| 16:0 | - ve | PI 255.3 *m/z* | 45 |
| 18:2 | - ve | PI 279.3 *m/z* | 45 |
| 18:1 | - ve | PI 281.3 *m/z* | 45 |
| 18:0 | - ve | PI 283.3 *m/z* | 45 |
