# Supplementary material for: Serum-Induced Keratinization Processes in an Immortalized Human Meibomian Gland Epithelial Cell Line
Source: PLoS One. 2015 Jun 4;10(6):e0128096. doi: 10.1371/journal.pone.0128096 (PMC4456149; doi:10.1371/journal.pone.0128096)
Supplement: S2 Table — All measurements are listed as mol% of total lipid. (DOCX) [file pone.0128096.s004.docx]

**Serum-induced keratinization processes of human meibomian gland epithelial cells**

Ulrike Hampel; Antje Schröder; Todd Mitchell; Simon Brown; Peta Snikeris; Fabian Garreis; Carolina Kunnen; Mark Willcox; Friedrich Paulsen

**S2 Table.** Lipid class means and standard error (n=15) in HMGEC cultivated for 1 day or 3 days in serum-containing medium. All measurements are listed as mol% of total lipid.

| Lipid Species | 1 day | | 3 days | |
| --- | --- | --- | --- | --- |
|  | **Mean (mol%)** | **SEM** | **Mean (mol%)** | **SEM** |
| PC | 35 | 2 | 28 | 2 |
| FC | 27 | 1 | 29 | 2 |
| PS | 12 | 3 | 12 | 4 |
| PE | 11 | 1 | 9.5 | 0.6 |
| CE | 4.4 | 0.5 | 4.3 | 0.5 |
| SM | 4.8 | 0.2 | 5.2 | 0.3 |
| DAG | 3.9 | 0.4 | 5.0 | 0.7 |
| TAG | 2.5 | 0.3 | 5.4 | 0.8 |
| Cer | 0.37 | 0.04 | 0.83 | 0.14 |
| WE | 0.46 | 0.11 | 0.35 | 0.10 |
