# Supplementary material for: Serum-Induced Keratinization Processes in an Immortalized Human Meibomian Gland Epithelial Cell Line
Source: PLoS One. 2015 Jun 4;10(6):e0128096. doi: 10.1371/journal.pone.0128096 (PMC4456149; doi:10.1371/journal.pone.0128096)
Supplement: S3 Table — All measurements are listed as mol% of total lipid. (DOCX) [file pone.0128096.s005.docx]

**Serum-induced keratinization processes of human meibomian gland epithelial cells**

Ulrike Hampel; Antje Schröder; Todd Mitchell; Simon Brown; Peta Snikeris; Fabian Garreis; Carolina Kunnen; Mark Willcox; Friedrich Paulsen

**Supporting information**

**S3 Table S5.** CE molecular lipid means and standard error (n=15) in HMGEC cultivated for 1 day or 3 days in serum-containing medium. All measurements are listed as mol% of total lipid.

| Lipid Species | 1 day | | 3 days | |
| --- | --- | --- | --- | --- |
|  | **Mean (mol%)** | **SEM** | **Mean (mol%)** | **SEM** |
| CE 14:0 | 0.062 | 0.006 | 0.063 | 0.009 |
| CE 15:0 | 0.024 | 0.005 | 0.023 | 0.005 |
| CE 16:0 | 0.183 | 0.018 | 0.189 | 0.018 |
| CE 16:1 | 0.113 | 0.010 | 0.114 | 0.011 |
| CE 17:0 | 0.033 | 0.005 | 0.034 | 0.004 |
| CE 17:1 | 0.018 | 0.003 | 0.018 | 0.003 |
| CE 18:0 | 0.053 | 0.007 | 0.083 | 0.009 |
| CE 18:1 | 0.462 | 0.037 | 0.406 | 0.030 |
| CE 18:2 | 0.084 | 0.009 | 0.072 | 0.005 |
| CE 19:0 | 0.058 | 0.008 | 0.049 | 0.006 |
| CE 19:1 | 0.011 | 0.002 | 0.009 | 0.002 |
| CE 20:0 | 0.064 | 0.008 | 0.064 | 0.008 |
| CE 20:1 | 0.038 | 0.003 | 0.041 | 0.005 |
| CE 20:2 | 0.042 | 0.004 | 0.040 | 0.004 |
| CE 20:3 | 0.048 | 0.004 | 0.057 | 0.006 |
| CE 20:4 | 0.062 | 0.009 | 0.107 | 0.015 |
| CE 21:0 | 0.040 | 0.006 | 0.039 | 0.005 |
| CE 21:1 | 0.228 | 0.029 | 0.202 | 0.022 |
| CE 22:0 | 0.025 | 0.004 | 0.026 | 0.004 |
| CE 22:1 | 0.013 | 0.002 | 0.016 | 0.003 |
| CE 22:2 | 0.012 | 0.002 | 0.016 | 0.002 |
| CE 23:0 | 0.040 | 0.005 | 0.036 | 0.006 |
| CE 23:1 | 0.002 | 0.001 | 0.002 | 0.001 |
| CE 23:2 | 0.002 | 0.001 | 0.001 | 0.001 |
| CE 24:0 | 0.133 | 0.016 | 0.134 | 0.017 |
| CE 24:1 | 1.868 | 0.231 | 1.743 | 0.178 |
| CE 24:2 | 0.014 | 0.002 | 0.014 | 0.002 |
| CE 25:0 | 0.040 | 0.006 | 0.036 | 0.006 |
| CE 25:1 | 0.003 | 0.002 | 0.004 | 0.002 |
| CE 25:2 | 0.001 | 0.001 | 0.002 | 0.001 |
| CE 26:0 | 0.024 | 0.005 | 0.023 | 0.004 |
| CE 26:1 | 0.052 | 0.005 | 0.053 | 0.005 |
| CE 26:2 | 0.015 | 0.003 | 0.016 | 0.002 |
| CE 27:0 | 0.058 | 0.010 | 0.045 | 0.005 |
| CE 27:1 | 0.011 | 0.002 | 0.014 | 0.003 |
| CE 27:2 | 0.003 | 0.001 | 0.002 | 0.001 |
| CE 27:3 | 0.173 | 0.017 | 0.236 | 0.021 |
| CE 28:0 | 0.008 | 0.003 | 0.006 | 0.002 |
| CE 28:1 | 0.011 | 0.003 | 0.019 | 0.003 |
| CE 28:2 | 0.004 | 0.001 | 0.002 | 0.001 |
| CE 29:0 | 0.002 | 0.001 | 0.002 | 0.001 |
| CE 29:1 | 0.003 | 0.002 | 0.003 | 0.002 |
| CE 29:2 | 0.004 | 0.002 | 0.003 | 0.001 |
| CE 29:3 | 0.016 | 0.003 | 0.021 | 0.003 |
| CE 30:0 | 0.002 | 0.001 | 0.000 | 0.000 |
| CE 30:1 | 0.031 | 0.005 | 0.034 | 0.004 |
| CE 30:2 | 0.008 | 0.002 | 0.010 | 0.002 |
| CE 31:0 | 0.010 | 0.003 | 0.006 | 0.002 |
| CE 31:1 | 0.005 | 0.002 | 0.006 | 0.002 |
| CE 32:0 | 0.005 | 0.002 | 0.005 | 0.001 |
| CE 32:1 | 0.067 | 0.008 | 0.074 | 0.007 |
| CE 32:2 | 0.013 | 0.003 | 0.016 | 0.003 |
| CE 33:0 | 0.008 | 0.002 | 0.006 | 0.002 |
| CE 34:0 | 0.005 | 0.001 | 0.004 | 0.002 |
| CE 34:1 | 0.012 | 0.002 | 0.016 | 0.003 |
| CE 34:2 | 0.030 | 0.005 | 0.028 | 0.004 |
| Total CE | 4.4 | 0.5 | 4.3 | 0.5 |
