# Supplementary material for: Serum-Induced Keratinization Processes in an Immortalized Human Meibomian Gland Epithelial Cell Line
Source: PLoS One. 2015 Jun 4;10(6):e0128096. doi: 10.1371/journal.pone.0128096 (PMC4456149; doi:10.1371/journal.pone.0128096)
Supplement: S4 Table — All measurements are listed as mol% of total lipid. (DOCX) [file pone.0128096.s006.docx]

**Serum-induced keratinization processes of human meibomian gland epithelial cells**

Ulrike Hampel; Antje Schröder; Todd Mitchell; Simon Brown; Peta Snikeris; Fabian Garreis; Carolina Kunnen; Mark Willcox; Friedrich Paulsen

**Supporting information**

**S4 Table.** PL molecular lipid means and standard error (n=15) in HMGEC cultivated for 1 day or 3 days in serum-containing medium. All measurements are listed as mol% of total lipid.

| Lipid Species | 1 day | | 3 days | |
| --- | --- | --- | --- | --- |
|  | **Mean (mol%)** | **SEM** | **Mean (mol%)** | **SEM** |
| PC 30:0 | 0.894 | 0.037 | 0.462 | 0.045 |
| PC 32:0 | 2.726 | 0.143 | 2.122 | 0.148 |
| PC 32:1 | 2.195 | 0.104 | 1.410 | 0.123 |
| PC 32:2 | 0.280 | 0.019 | 0.194 | 0.023 |
| PC 34:1 | 7.355 | 0.276 | 5.713 | 0.380 |
| PC 34:2 | 2.003 | 0.113 | 1.390 | 0.159 |
| PC 34:3 | 0.194 | 0.011 | 0.155 | 0.017 |
| PC 36:0 | 0.145 | 0.014 | 0.265 | 0.052 |
| PC 36:1 | 1.790 | 0.099 | 2.946 | 0.216 |
| PC 36:2 | 5.027 | 0.208 | 3.491 | 0.327 |
| PC 36:3 | 0.719 | 0.031 | 0.535 | 0.049 |
| PC 36:4 | 0.195 | 0.011 | 0.261 | 0.025 |
| PC 36:5 | 0.305 | 0.035 | 0.421 | 0.050 |
| PC 38:1 | 0.112 | 0.009 | 0.185 | 0.026 |
| PC 38:2 | 0.373 | 0.018 | 0.259 | 0.019 |
| PC 38:3 | 0.608 | 0.030 | 0.322 | 0.023 |
| PC 38:4 | 0.235 | 0.012 | 0.232 | 0.016 |
| PC 38:5 | 0.508 | 0.064 | 0.796 | 0.105 |
| PC 38:6 | 0.911 | 0.101 | 1.172 | 0.151 |
| PC 40:2 | 0.043 | 0.003 | 0.050 | 0.005 |
| PC 40:3 | 0.073 | 0.004 | 0.065 | 0.005 |
| PC 40:4 | 0.094 | 0.004 | 0.072 | 0.005 |
| PC 40:5 | 0.118 | 0.011 | 0.205 | 0.030 |
| PC 40:6 | 0.414 | 0.038 | 0.975 | 0.124 |
| PC O-30:0 | 0.294 | 0.018 | 0.141 | 0.015 |
| PC O-32:0 | 0.829 | 0.087 | 0.290 | 0.023 |
| PC O-32:1 | 0.537 | 0.044 | 0.347 | 0.031 |
| PC O-34:0 | 0.092 | 0.009 | 0.067 | 0.005 |
| PC O-34:1 | 1.449 | 0.145 | 0.653 | 0.070 |
| PC O-34:2 | 0.548 | 0.029 | 0.560 | 0.044 |
| PC O-36:0 | 0.042 | 0.003 | 0.029 | 0.004 |
| PC O-36:1 | 0.239 | 0.016 | 0.225 | 0.014 |
| PC O-36:2 | 0.573 | 0.048 | 0.380 | 0.038 |
| PC O-36:3 | 0.261 | 0.014 | 0.236 | 0.020 |
| PC O-36:4 | 0.118 | 0.007 | 0.081 | 0.007 |
| PC O-36:5 | 0.285 | 0.033 | 0.156 | 0.020 |
| PC O-38:1 | 0.073 | 0.007 | 0.057 | 0.004 |
| PC O-38:2 | 0.102 | 0.007 | 0.073 | 0.006 |
| PC O-38:3 | 0.128 | 0.016 | 0.060 | 0.007 |
| PC O-38:4 | 0.115 | 0.007 | 0.069 | 0.008 |
| PC O-38:5 | 0.219 | 0.019 | 0.159 | 0.018 |
| PC O-38:6 | 0.658 | 0.056 | 0.473 | 0.058 |
| PC O-40:3 | 0.044 | 0.006 | 0.038 | 0.005 |
| PC O-40:4 | 0.055 | 0.005 | 0.026 | 0.005 |
| PC O-40:5 | 0.048 | 0.004 | 0.044 | 0.006 |
| PC O-40:6 | 0.217 | 0.018 | 0.221 | 0.017 |
| PC O-40:7 | 0.435 | 0.048 | 0.379 | 0.046 |
| Total PC | 34.67 | 0.037 | 0.462 | 0.045 |
| PE 32:0 | 0.038 | 0.143 | 2.122 | 0.148 |
| PE 32:1 | 0.082 | 0.104 | 1.410 | 0.123 |
| PE 34:1 | 0.619 | 0.019 | 0.194 | 0.023 |
| PE 34:2 | 0.245 | 0.276 | 5.713 | 0.380 |
| PE 34:3 | 0.003 | 0.113 | 1.390 | 0.159 |
| PE 36:1 | 1.073 | 0.011 | 0.155 | 0.017 |
| PE 36:2 | 1.512 | 0.014 | 0.265 | 0.052 |
| PE 36:3 | 0.209 | 0.099 | 2.946 | 0.216 |
| PE 36:4 | 0.022 | 0.208 | 3.491 | 0.327 |
| PE 36:5 | 0.030 | 0.031 | 0.535 | 0.049 |
| PE 38:0 | 0.085 | 0.011 | 0.261 | 0.025 |
| PE 38:1 | 0.126 | 0.035 | 0.421 | 0.050 |
| PE 38:2 | 0.239 | 0.009 | 0.185 | 0.026 |
| PE 38:3 | 0.344 | 0.018 | 0.259 | 0.019 |
| PE 38:4 | 0.220 | 0.030 | 0.322 | 0.023 |
| PE 38:5 | 0.305 | 0.012 | 0.232 | 0.016 |
| PE 38:6 | 0.293 | 0.064 | 0.796 | 0.105 |
| PE 40:1 | 0.022 | 0.101 | 1.172 | 0.151 |
| PE 40:2 | 0.066 | 0.003 | 0.050 | 0.005 |
| PE 40:3 | 0.091 | 0.004 | 0.065 | 0.005 |
| PE 40:4 | 0.107 | 0.004 | 0.072 | 0.005 |
| PE 40:5 | 0.187 | 0.011 | 0.205 | 0.030 |
| PE 40:6 | 0.580 | 0.038 | 0.975 | 0.124 |
| PE 40:7 | 0.556 | 0.018 | 0.141 | 0.015 |
| PE 40:8 | 0.026 | 0.087 | 0.290 | 0.023 |
| PE O-34:0 | 0.047 | 0.044 | 0.347 | 0.031 |
| PE O-34:1 | 0.015 | 0.009 | 0.067 | 0.005 |
| PE O-34:2 | 0.470 | 0.145 | 0.653 | 0.070 |
| PE O-36:1 | 0.188 | 0.029 | 0.560 | 0.044 |
| PE O-36:2 | 0.259 | 0.003 | 0.029 | 0.004 |
| PE O-36:3 | 0.383 | 0.016 | 0.225 | 0.014 |
| PE O-36:4 | 0.037 | 0.048 | 0.380 | 0.038 |
| PE O-36:5 | 0.082 | 0.014 | 0.236 | 0.020 |
| PE O-38:0 | 0.125 | 0.007 | 0.081 | 0.007 |
| PE O-38:1 | 0.010 | 0.033 | 0.156 | 0.020 |
| PE O-38:2 | 0.057 | 0.007 | 0.057 | 0.004 |
| PE O-38:3 | 0.113 | 0.007 | 0.073 | 0.006 |
| PE O-38:4 | 0.192 | 0.016 | 0.060 | 0.007 |
| PE O-38:5 | 0.211 | 0.007 | 0.069 | 0.008 |
| PE O-38:6 | 1.011 | 0.019 | 0.159 | 0.018 |
| PE O-40:3 | 0.022 | 0.056 | 0.473 | 0.058 |
| PE O-40:4 | 0.039 | 0.006 | 0.038 | 0.005 |
| PE O-40:5 | 0.095 | 0.005 | 0.026 | 0.005 |
| PE O-40:6 | 0.183 | 0.004 | 0.044 | 0.006 |
| PE | 10.61 | 0.56 | 9.54 | 0.57 |
| PS 36:1 | 6.562 | 1.372 | 6.465 | 1.386 |
| PS 36:2 | 0.164 | 0.045 | 0.500 | 0.166 |
| PS 36:3 | 0.361 | 0.070 | 0.288 | 0.075 |
| PS 38:1 | 0.322 | 0.069 | 0.199 | 0.041 |
| PS 38:2 | 0.660 | 0.128 | 0.211 | 0.087 |
| PS 38:3 | 0.091 | 0.027 | 0.136 | 0.055 |
| PS 38:4 | 0.157 | 0.033 | 0.193 | 0.054 |
| PS 38:5 | 0.046 | 0.035 | 0.004 | 0.003 |
| PS 40:1 | 0.227 | 0.050 | 0.259 | 0.051 |
| PS 40:2 | 0.457 | 0.079 | 0.252 | 0.083 |
| PS 40:3 | 0.334 | 0.059 | 0.183 | 0.051 |
| PS 40:4 | 0.242 | 0.042 | 0.115 | 0.046 |
| PS 40:5 | 0.818 | 0.231 | 1.110 | 0.359 |
| PS 40:6 | 0.922 | 0.252 | 1.478 | 0.561 |
| PS O-36:1 | 0.117 | 0.028 | 0.170 | 0.052 |
| PS O-36:2 | 0.386 | 0.097 | 0.473 | 0.131 |
| Total PS | 11.87 | 3.16 | 12.04 | 3.63 |
| SM 32:1;2 | 0.160 | 0.006 | 0.141 | 0.008 |
| SM 33:0;2 | 0.000 | 0.000 | 0.000 | 0.000 |
| SM 33:1;2 | 0.060 | 0.006 | 0.047 | 0.006 |
| SM 34:0;2 | 0.584 | 0.044 | 1.142 | 0.056 |
| SM 34:1;2 | 2.600 | 0.093 | 2.156 | 0.071 |
| SM 34:2;2 | 0.138 | 0.007 | 0.092 | 0.006 |
| SM 35:0;2 | 0.000 | 0.000 | 0.000 | 0.000 |
| SM 35:1;2 | 0.000 | 0.000 | 0.001 | 0.001 |
| SM 36:1;2 | 0.000 | 0.000 | 0.000 | 0.000 |
| SM 36:2;2 | 0.000 | 0.000 | 0.000 | 0.000 |
| SM 41:0;2 | 0.000 | 0.000 | 0.000 | 0.000 |
| SM 41:1;2 | 0.000 | 0.000 | 0.000 | 0.000 |
| SM 41:2;2 | 0.000 | 0.000 | 0.002 | 0.002 |
| SM 42:0;2 | 0.003 | 0.001 | 0.065 | 0.015 |
| SM 42:1;2 | 0.331 | 0.018 | 0.580 | 0.037 |
| SM 42:2;2 | 0.592 | 0.024 | 0.610 | 0.040 |
| SM 43:0;2 | 0.000 | 0.000 | 0.000 | 0.000 |
| SM 43:1;2 | 0.001 | 0.001 | 0.001 | 0.001 |
| SM 43:2;2 | 0.000 | 0.000 | 0.000 | 0.000 |
| SM 44:0;2 | 0.000 | 0.000 | 0.000 | 0.000 |
| SM 44:1;2 | 0.105 | 0.008 | 0.148 | 0.015 |
| SM 44:2;2 | 0.167 | 0.009 | 0.170 | 0.012 |
| SM 44:3;2 | 0.004 | 0.003 | 0.004 | 0.003 |
| Total SM | 4.75 | 0.24 | 5.16 | 0.28 |
