# Supplementary material for: Serum-Induced Keratinization Processes in an Immortalized Human Meibomian Gland Epithelial Cell Line
Source: PLoS One. 2015 Jun 4;10(6):e0128096. doi: 10.1371/journal.pone.0128096 (PMC4456149; doi:10.1371/journal.pone.0128096)
Supplement: S5 Table — All measurements are listed as mol% of total lipid. (DOCX) [file pone.0128096.s007.docx]

**Serum-induced keratinization processes of human meibomian gland epithelial cells**

Ulrike Hampel; Antje Schröder; Todd Mitchell; Simon Brown; Peta Snikeris; Fabian Garreis; Carolina Kunnen; Mark Willcox; Friedrich Paulsen

**Supporting information**

**S5 Table.** DAG and TAG molecular lipids, mean and standard error (n=15) in HMGEC cultivated for 1 day or 3 days in serum-containing medium. All measurements are listed as mol% of total lipid.

| Lipid Species | 1 day | | 3 days | |
| --- | --- | --- | --- | --- |
|  | **Mean (mol%)** | **SEM** | **Mean (mol%)** | **SEM** |
| DAG 32:0 | 0.000 | 0.000 | 0.000 | 0.000 |
| DAG 32:1 | 0.031 | 0.006 | 0.025 | 0.006 |
| DAG 32:2 | 0.007 | 0.002 | 0.002 | 0.001 |
| DAG 34:0 | 0.220 | 0.024 | 0.337 | 0.031 |
| DAG 34:1 | 0.873 | 0.077 | 1.031 | 0.094 |
| DAG 34:2 | 0.863 | 0.080 | 0.806 | 0.089 |
| DAG 34:3 | 0.036 | 0.005 | 0.023 | 0.006 |
| DAG 36:0 | 0.169 | 0.025 | 0.222 | 0.026 |
| DAG 36:1 | 0.316 | 0.030 | 0.585 | 0.059 |
| DAG 36:2 | 0.692 | 0.053 | 0.789 | 0.078 |
| DAG 36:3 | 0.111 | 0.011 | 0.131 | 0.015 |
| DAG 36:4 | 0.016 | 0.005 | 0.046 | 0.008 |
| DAG 38:4 | 0.102 | 0.014 | 0.240 | 0.029 |
| DAG 38:5 | 0.153 | 0.015 | 0.175 | 0.021 |
| DAG 40:5 | 0.106 | 0.013 | 0.187 | 0.023 |
| DAG 40:6 | 0.208 | 0.048 | 0.410 | 0.091 |
| Total DAG | 3.90 | 0.41 | 5.01 | 0.69 |
| TAG 48:0 | 0.035 | 0.008 | 0.101 | 0.013 |
| TAG 48:1 | 0.079 | 0.005 | 0.197 | 0.023 |
| TAG 48:2 | 0.051 | 0.004 | 0.105 | 0.013 |
| TAG 48:3 | 0.015 | 0.002 | 0.023 | 0.003 |
| TAG 50:0 | 0.034 | 0.010 | 0.120 | 0.014 |
| TAG 50:1 | 0.194 | 0.015 | 0.591 | 0.073 |
| TAG 50:2 | 0.179 | 0.013 | 0.412 | 0.048 |
| TAG 50:3 | 0.102 | 0.007 | 0.166 | 0.016 |
| TAG 50:4 | 0.013 | 0.002 | 0.022 | 0.003 |
| TAG 52:0 | 0.017 | 0.008 | 0.052 | 0.007 |
| TAG 52:1 | 0.075 | 0.005 | 0.257 | 0.030 |
| TAG 52:2 | 0.514 | 0.038 | 1.123 | 0.130 |
| TAG 52:3 | 0.273 | 0.023 | 0.477 | 0.053 |
| TAG 52:4 | 0.031 | 0.003 | 0.081 | 0.011 |
| TAG 52:5 | 0.006 | 0.001 | 0.017 | 0.003 |
| TAG 54:0 | 0.003 | 0.002 | 0.006 | 0.001 |
| TAG 54:1 | 0.029 | 0.003 | 0.146 | 0.018 |
| TAG 54:2 | 0.221 | 0.014 | 0.581 | 0.064 |
| TAG 54:3 | 0.433 | 0.036 | 0.662 | 0.073 |
| TAG 54:4 | 0.124 | 0.011 | 0.209 | 0.023 |
| TAG 54:5 | 0.031 | 0.004 | 0.070 | 0.009 |
| TAG 54:6 | 0.001 | 0.001 | 0.005 | 0.001 |
| Total TAG | 2.46 | 0.25 | 5.42 | 0.83 |
