# Supplementary material for: Serum-Induced Keratinization Processes in an Immortalized Human Meibomian Gland Epithelial Cell Line
Source: PLoS One. 2015 Jun 4;10(6):e0128096. doi: 10.1371/journal.pone.0128096 (PMC4456149; doi:10.1371/journal.pone.0128096)
Supplement: S6 Table — All measurements are listed as mol% of total lipid. (DOCX) [file pone.0128096.s008.docx]

**Serum-induced keratinization processes of human meibomian gland epithelial cells**

Ulrike Hampel; Antje Schröder; Todd Mitchell; Simon Brown; Peta Snikeris; Fabian Garreis; Carolina Kunnen; Mark Willcox; Friedrich Paulsen

**Supporting information**

**S6 Table.** WE molecular lipids, mean and standard error (n=15) in HMGEC cultivated for 1 day or 3 days in serum-containing medium. All measurements are listed as mol% of total lipid.

| Lipid Species | 1 day | | | 3 days | | |
| --- | --- | --- | --- | --- | --- | --- |
|  | **Mean (mol%)** | **SEM** | **Mean (mol%)** | | **SEM** |  |
| WE_20:0-O/ 16:0 | 0.010 | 0.003 | 0.007 | | 0.004 |  |
| WE_20:0-O/ 16:1 | 0.017 | 0.009 | 0.021 | | 0.017 |  |
| WE_20:0-O/ 17:0 | 0.002 | 0.001 | 0.002 | | 0.001 |  |
| WE_20:0-O/ 18:1 | 0.041 | 0.011 | 0.025 | | 0.006 |  |
| WE_21:0-O/ 16:0 | 0.003 | 0.001 | 0.005 | | 0.002 |  |
| WE_21:0-O/ 16:1 | 0.010 | 0.006 | 0.010 | | 0.009 |  |
| WE_21:0-O/ 17:0 | 0.002 | 0.001 | 0.000 | | 0.000 |  |
| WE_21:0-O/ 18:1 | 0.034 | 0.009 | 0.013 | | 0.004 |  |
| WE_22:0-O/ 16:0 | 0.005 | 0.002 | 0.004 | | 0.002 |  |
| WE_22:0-O/ 16:1 | 0.011 | 0.006 | 0.010 | | 0.008 |  |
| WE_22:0-O/ 17:0 | 0.008 | 0.004 | 0.011 | | 0.004 |  |
| WE_22:0-O/ 18:1 | 0.042 | 0.009 | 0.030 | | 0.009 |  |
| WE_23:0-O/ 16:0 | 0.000 | 0.000 | 0.000 | | 0.000 |  |
| WE_23:0-O/ 16:1 | 0.031 | 0.011 | 0.017 | | 0.009 |  |
| WE_23:0-O/ 17:0 | 0.024 | 0.003 | 0.040 | | 0.003 |  |
| WE_23:0-O/ 18:1 | 0.109 | 0.014 | 0.063 | | 0.011 |  |
| WE_24:0-O/ 16:0 | 0.010 | 0.004 | 0.005 | | 0.003 |  |
| WE_24:0-O/ 16:1 | 0.001 | 0.001 | 0.006 | | 0.005 |  |
| WE_24:0-O/ 17:0 | 0.003 | 0.001 | 0.003 | | 0.002 |  |
| WE_24:0-O/ 18:1 | 0.017 | 0.007 | 0.007 | | 0.004 |  |
| WE_25:0-O/ 16:0 | 0.001 | 0.001 | 0.003 | | 0.001 |  |
| WE_25:0-O/ 16:1 | 0.001 | 0.001 | 0.001 | | 0.001 |  |
| WE_25:0-O/ 17:0 | 0.005 | 0.001 | 0.005 | | 0.002 |  |
| WE_25:0-O/ 18:1 | 0.015 | 0.005 | 0.010 | | 0.004 |  |
| WE_26:0-O/ 16:0 | 0.004 | 0.002 | 0.002 | | 0.001 |  |
| WE_26:0-O/ 16:1 | 0.002 | 0.001 | 0.000 | | 0.000 |  |
| WE_26:0-O/ 17:0 | 0.000 | 0.000 | 0.000 | | 0.000 |  |
| WE_26:0-O/ 18:1 | 0.009 | 0.006 | 0.002 | | 0.001 |  |
| WE_27:0-O/ 16:0 | 0.000 | 0.000 | 0.000 | | 0.000 |  |
| WE_27:0-O/ 16:1 | 0.000 | 0.000 | 0.001 | | 0.001 |  |
| WE_27:0-O/ 17:0 | 0.003 | 0.002 | 0.001 | | 0.001 |  |
| WE_27:0-O/ 18:1 | 0.008 | 0.003 | 0.015 | | 0.005 |  |
| WE_28:0-O/ 16:0 | 0.005 | 0.003 | 0.001 | | 0.001 |  |
| WE_28:0-O/ 16:1 | 0.008 | 0.007 | 0.000 | | 0.000 |  |
| WE_28:0-O/ 17:0 | 0.001 | 0.001 | 0.000 | | 0.000 |  |
| WE_28:0-O/ 18:1 | 0.006 | 0.003 | 0.011 | | 0.004 |  |
| WE_29:0-O/ 16:0 | 0.000 | 0.000 | 0.000 | | 0.000 |  |
| WE_29:0-O/ 16:1 | 0.000 | 0.000 | 0.013 | | 0.009 |  |
| WE_29:0-O/ 17:0 | 0.001 | 0.000 | 0.000 | | 0.000 |  |
| WE_29:0-O/ 18:1 | 0.004 | 0.003 | 0.002 | | 0.001 |  |
| WE_29:0-O/ 16:0 | 0.010 | 0.003 | 0.007 | | 0.004 |  |
| WE_29:0-O/ 16:1 | 0.017 | 0.009 | 0.021 | | 0.017 |  |
| WE_29:0-O/ 17:0 | 0.002 | 0.001 | 0.002 | | 0.001 |  |
| WE_29:0-O/ 18:1 | 0.041 | 0.011 | 0.025 | | 0.006 |  |
| Total WE | 0.46 | 0.11 | 0.35 | | 0.10 |  |
